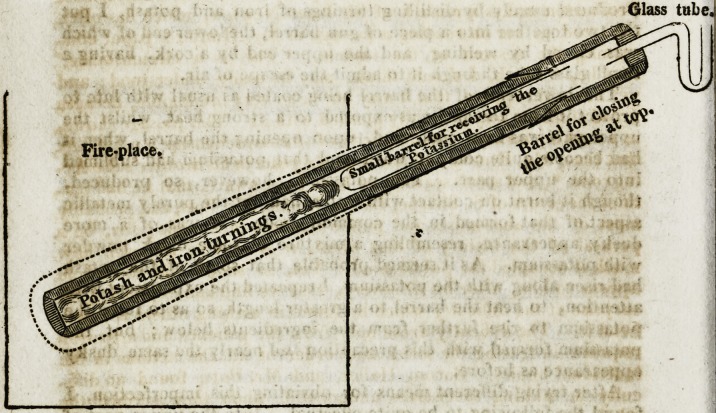# Collectanea Medica, Consisting of Anecdotes, Facts, Extracts, Illustrations, Queries, Suggestions, &c.

**Published:** 1815-07

**Authors:** 


### COLLECTANEA MEDICA,

CONSISTING OF

### ANECDOTES, FACTS, EXTRACTS, ILLUSTRATIONS, QUERIES, SUGGESTIONS, &c.

RELATING TO THE

*History or the Art of Medicine, and the Auxiliary Sciences.*

---

### *On an easier Mode of procuring Potassium than that which is now adopted.* By SMITHSON TENNANT, Esq. F.R.S.

THE great discovery of Sir H. Davy, that the alkalies might be decomposed by the Voltaic electricity, was soon succeeded by that of Gay Lussac and Thenard, who showed that a similar decomposition of them might be produced by means of iron.

Besides the new and unexpected fact which was thus brought to light, that the alkaline metals might be deprived of oxygen by a substance inferior to them in attraction; this new process was highly valuable, in affording the means of obtaining them far more abundantly than by electricity.

The circumstances described by Gay Lussac and Thenard, as requisite for producing the decomposition of the alkalies by iron, are, first, that the iron should be intensely heated, and afterwards, that the alkalies should be brought in contact with it in that heated state. For this purpose, a furnace must be made, capable of admitting a gun barrel, containing the iron turnings, to pass through it, and a short piece of barrel containing the alkali must be adapted to the former by grinding, so as to be air tight. As this short piece of barrel is out of the furnace, G. Lussac and Thenard direct that a separate fire be applied to it, in order to make the alkali pass from it into the longer barrel. To avoid the necessity of a separate fire, this passage of the alkali has, in England, been generally effected through a small perforation between the two barrels, being poured very hot into the smaller barrel, which is then closed with a ground stopper.

The process conducted in either way, requires the construction of a particular furnace, and the correct fitting of the barrels by grinding, so as to be air-tight, and, being somewhat complicated, is not always performed with success.

As it was very desirable to facilitate the mode of obtaining potassium, which is so powerful a chemical agent, I have attempted to simplify the process, and, having so far succeeded as to render it capable of being performed in a common smith's fire, and without the junction of the iron barrels by grinding, I have thought it might deserve to be communicated to the Royal Society.

If

If it was absolutely necessary to heat the alkali and iron separately, and in that state to unite them, no material improvement in the simplicity of the present apparatus could be reasonably looked for; but, upon considering that the alkali frequently passed through the short barrel in a few minutes, it did not seem probable that much of the potassium was then formed, since the whole operation required a continuance of the heat for near an hour.

In order, therefore, to learn whether potassium might not be produced merely by distilling turnings of iron and potash, I put the two together into a piece of gun barrel, the lower end of which was closed by welding, and the upper end by a cork, having a small glass tube through it to admit the escape of air.

The lower end of the barrel being coated as usual with lute to protect it from the air, was exposed to a strong heat, whilst the upper part was kept cool, and, upon opening the barrel, when it had become quite cold, it was found that potassium had sublimed into the upper part. The potassium, however, so produced, though it burnt on contact with water, had not the purely metallic aspect of that formed in the common mode. It was of a more dusky appearance, resembling a mixture of some black powder with potassium. As it seemed probable that some of the potash had risen along with the potassium, I repeated the experiment with attention, to heat the barrel to a greater length, so as to force the potassium to rise further from the ingredients below; but the potassium formed with this precaution had nearly the same dusky appearance as before.

After trying different means for obviating this imperfection, I found the following to be quite effectual. Into the upper part of the barrel, a narrower piece, nearly fitting it, was inserted, open only by a perforation at the lower end to admit the vapour of the potassium to pass into it, and, upon distilling potash and iron turnings with this addition, the potassium rose into the narrow tube, quite pure, with its usual brightness.

The most convenient dimensions of the apparatus are for the external barrel to be about a foot and a half long, and the internal one about seven or eight inches. The latter should not be wholly inserted in the former, but about an inch of it left out for the greater ease in withdrawing it. The width is in general determined by that of a common gun barrel, but may be increased to a certain degree. I have had the thick part of a gun barrel so much enlarged by hammering it thinner, as to contain twice as much iron turnings and potash, and have employed it with success. But, on the other hand, there are limits to this extension of the width, arising from the increased difficulty of making the heat penetrate throughout.

The opening of the barrels at the top must be covered with a cap or wide tube, which, being at a distance from the fire, need only be fastened with sealing wax; but, for the greater security of keeping this part cool, the whole of the tube which is out of the fire should be wrapped round with linen or blotting paper kept wet.

The opening of the wide tube must be closed with a cork, having a crooked tube of glass through it, containing a drop of mercury, which, being moved by the passage of the air, shews that the vessels are perfectly tight. But the annexed sketch will at once show both the construction and dimensions of the apparatus.

The principal point to be attended to, both in this and in the common mode, is the giving a strong heat, which should be continued for the greater part of an hour; and, to enable the iron barrel to support this, it is quite essential to cover it with a proper lute, carefully applied.

The lute which I have found most effectual for this purpose, was composed of a small proportion of Stourbridge clay in its natural state, with a much larger proportion which had before been burnt and powdered, and both of which may be easily had at any glass-house.—*Phil. Trans.*

Our next Number will contain a very useful paper on the Economy of Heat, also by Smithson Tenant, Esq.—probably the last from that much-lamented philosopher.

---

The following is from a pamphlet written in answer to a criticism on a work we have already reviewed. To avoid hyper-criticism, therefore, if our readers will admit such a pun, we confine ourselves to extracts, and insert them in our *Collectanea.*

Apology

***Apology for Mr. Hunter's supposed Obscurity, and illustration of his Doctrine concerning the Vitality of the Blood.—From Dr. Adams's Answer to the Edinburgh Reviewers.***

"In other sciences also, (says Dr. Adams,) are not students expected to apply with diligence to those authors, a clear comprehension of whose writings is considered as indispensable to their further progress? Nor is it surprising, that superior minds should not, at all times, see the train of observation by which they arrived at their conclusions. Pemberton makes a remark of Sir Isaac Newton, which has often occurred to me on reading a passage in Mr. Hunter's paper concerning the descent of the testicle. 'This is a circumstance,' says he, 'which, I should think, may be easily understood; and yet I should suppose, that it may not be so very intelligible, because I find students very generally puzzled with it.' This passage shows, in a striking manner, the simplicity of so comprehensive a mind. At that time, Mr. Hunter had not been six years in his brother's dissecting room *, and seems scarcely to have been aware, that he had rectified the errors of S. Sharpe, Bacon, Haller, Mr. Pott, and probably most of the anatomists and public teachers in Europe; for, on a perusal of the history of that event, it is easy to discover how much it occupied the public mind. Yet, so simple did the whole appear to him, after his discovery, that we find him expressing his surprise at the difficulty which he found in making others comprehend it. The difficulty arose only from the patience and attention requisite in tracing the series and order of all the actions in the individual bodies. Bacon, Haller, and Mr. Pott, found no difficulty in making themselves understood, when they ascribed the whole to *causes* which could not exist, and which, if they had existed, would not have been equal to the general uniformity of the effect. Of this they were so sensible, that the first has paid the highest compliment to Mr. Hunter on his accuracy, † and Mr. Pott omitted the controversy in the subsequent edition of his works. Even Mr. Jesse Foote, who takes no pains to conceal his hostility to the Hunters, concludes this part of his history with the following sentence. 'I should not follow the bent of my inclination, nor comply with the command of my understanding, if I did not voluntarily own, that John Hunter, by those observations, has fairly obtained anatomical fame.'" Life of J. Hunter, p. 57.

---

* And was only described by Baron Haller as *Joannes* Hunter *Gulielmi* frater.—Ed.

† "Nos quidem testes in abdomine fœtus habitare, serius in "scrotum descendere vidimus, et aliquando peritonæum foramine "patuisse, per quod testis exiret. *Accuratius* hæc Johannes Hunter, "Gulielmi frater, exposuit, addidit, ut debilis cellulosa tela cedat, "testem transmittat, peritonæum vero supra transitum confirmet. "Hæc bonis iconibus exprimit." Halleri *Biblioth. Anatom.* tom. ii. p. 863.

This

"This history may serve to account for Mr. Hunter's supposed obscurity, without impeaching his accuracy.*

"The life of blood would probably never have been disputed, if Mr. Hunter had not made it part of his doctrine of inflammation. Harvey has devoted a long paper, not indeed to prove the life of blood, for that he seems to consider as admitted; but to show that its actions vary, according to the stimuli to which it is exposed, in the same manner as the actions of the solid parts. Harvey's physiology was directed to the generation of animals; Hunter's to pathology, and, first of all, to inflammation, as the most important inquiry in surgery. Harvey stood on the same ground as most discoverers. He demonstrated what was before conjectured. The fame he thus very justly acquired, induced others to dispute what was before unnoticed. But demonstration admitted of no dispute. Some, therefore, refused to look, and others refused his claim to originality.

"If the life of the blood was never disputed before Mr. Hunter's time, it was, because no one before him had traced with accuracy the progress of local actions under disease. In the prosecution of such an inquiry, it was found necessary to compare such actions with the customary actions of the same parts in health. As both depend on the same principle acting differently, according to the

---

* "Dr. Ley, in his elegant Inaugural Dissertation, (Edinb. 1813) de Pthisi Pulmonali, produces another illustration of the accuracy, yet apparent obscurity of Mr. Hunter. Speaking of hectic, as ascribed by Dr. Reid and Professor Cullen, to the absorption of pus, he concludes, 'Priusquam vero Culleni, ejusque sequacium, theoriam veram esse admittere possimus, necesse est ut demonstretur, imprimis, pus formari;—*2do*, hoc pus acre esse;—*3tio*, hoc pus acre absorberi;—et *4to*, hoc pus acre absorptum hecticae originem esse. Quoniam vero pluribus, ne dicam omnibus, in exemplis, hae res universae nullo modo accidere probatae sunt, haec opinio, ut mihi videtur, pro hypothesi spectarí debet. Altera de hecticae natura opinio, cujus mentionem feci, ea est a Johanne Huntero oblata. Haec autem opinio peculiari ejus dictione, ad quam suis in scriptis tantum adspiravit, direptâ, factorum solummodo est accumulatio. Hanc ejus opinionem ex commentis——Adams hic proferam: 'Hectic fever is an habitual, universal sympathy of the constitution struggling with a disease it is unable to overcome,' et nil dubium est ut in hoc morbo ita res se habeant. Haec igitur Hunteri sententia conclusio esse videtur permultis ex factis deducta; ob eamque causam, opinioni Culleni, quae hypothesin implicare videtur, multum anteponenda.'

"It was not necessary for the author to trace this opinion of the cause of the hectic fever beyond Cullen. It is, however, minutely dwelt on by Boerhaave; and his commentator, like most other good things, wishes to rest it upon the authority of Hippocrates. It was not only generally admitted before Mr. Hunter's time, but, like most other fables, was easily comprehended."

condition condition of the animal, it became necessary to trace, as minutely as possible, the principle itself. This led to the consideration of life, not merely as it appears in the whole animal, but as we discover it by its actions in every individual part.

"'I shall carry my ideas of life,' says Mr. Hunter, 'further than has generally been done. Life I believe to exist in every part of the animal; and to render it susceptible of impressions which excite action, there is no part which has not more or less of this principle: and, consequently, no part which does not act according to the nature of this principle itself, and the impressions thence arising, producing infinite variety, both in natural and diseased actions.' Introduct. p. 3. In other words, in order to direct our attention to the cure of local diseases, it will be necessary to trace carefully the actions of these parts in health, as well as under disease.

"In most diseased actions, perhaps in all connected with surgery, inflammation is that which requires the most immediate attention; and the appearance of the blood is, by common consent, considered as forming an important part in our decision. The consideration of the blood, therefore, and its changes, could not but attract the notice of Mr. Hunter. Others had examined it by the microscope, and by various chemical tests. These are not unnoticed by him; and it is worth observing, that whatever he has admitted in this part of the inquiry, has not been contradicted by subsequent researches. But none of these were sufficient to explain any of the changes which take place in the living body. The red particles, so minutely examined by the microscope, appear the least important part, as they are not to be met with in many animals; and artificial chemistry makes no part of coagulation the first change we discover in blood. To the immediate cause of this change, our attention is first directed, to the various forms under which it takes place, and to the causes which prevent it altogether. To what shall we ascribe this change? An apparently homogeneous fluid, spontaneously separating into a solid and a fluid part. Sometimes, we find a further separation of the solid part into two distinct portions, the red particles and the coagulated lymph; which last, in this case, appears opake, and of a complexion approaching the nearer to white, in proportion as the separation is more complete.

"'The first observations on the coagulation of blood,' says Mr. H. 'were probably made on animals whose heat was greater than the heat of the surrounding atmosphere. Hence coagulation was readily accounted for, by imputing it to cold. But that cold has no power of coagulating blood, has been proved in a variety of ways, and in none more pointedly than by Mr. Huson's experiments. He took blood, and froze it quickly: here it was in a state of congelation; but, when thawed, it recovered its fluidity, and soon afterwards coagulated.

"As blood, when out of the body, is exposed to the air, it was supposed that air might be the cause of its coagulation. But,

 if if such were the cause, we should find it uniformly follow such exposure to the air; on the contrary, under many circumstances, which will be explained hereafter, the blood never coagulates, though so exposed: under others, it coagulates within the body, and even within the vessels, where the external air cannot reach it.

"Rest is another cause, on which the coagulation of the blood was supposed to depend. It cannot be questioned, as far as we can trace the process of coagulation out of the body, that rest is favourable to it. But, if rest, unconnected with any other cause, were sufficient, we should find it uniformly follow such a condition of the blood; on the contrary, we find that the blood may rest or stagnate, in many parts of the body, without coagulating; and that, under certain circumstances, when there is no apparent impediment to its free circulation, it will coagulate in the vessels themselves. In priapism, it will not coagulate, though stagnant for a much longer time than it remains fluid out of the body; and, in cases of mortification, it will coagulate in the larger vessels, not from any impediment to the circulation, for, if that were the case, the same effect must follow in all cases of amputation.

"From all this, it follows, that none of the commonly assigned causes produce coagulation of the blood with any uniformity. When out of the body, we find this effect, indeed, usually produced; but neither constantly nor always in a similar form. The menstrual blood never coagulates; under certain modes of dying, the blood never coagulates; and there are conditions of life, under which blood taken from the veins never coagulates. Its form and mode of coagulating also varies. To what are we to impute this variety of appearances in the same fluid? If we find its actions correspond with the actions of the solid parts of the body, as we know that the actions of the latter depend on life, must we not infer the same in the blood? And if, with the loss of life, the capacity of action ceases in the solids, and we see the same incapacity to coagulation in the blood, under the same circumstances, should we not impute this also to the loss of life? And if we find that the blood coagulates in the body, or even in the vessels themselves, whenever its coagulation is necessary for the support or relief of a part, or the whole animal, that it remains fluid, when its solidity would be injurious to parts in which it stagnates, or to the whole body, if it should stagnate in the whole body; and if, with the entire loss of action, induced by death in the solid parts of the body, we find a similar incapacity in the blood to coagulate, whether in or out of the body, shall we not admit with Mr. Hunter, the harmony that exists between the actions of the living solids and of the blood, and impute both to the living principle? or, shall we prefer the language of Harvey, "eum (sanguinem) irritantis injuriam et foventis commodum persentiscere manifestum est, ideoque concludimus sanguinem per se vivere et nutriri."

"It may be objected, that the language of the discoverer of the circulation is somewhat figurative or poetical. This was too often the the error of the English writers of that date, and seems to have arisen from their great anxiety to render their Latin perfectly classical or Ciceronean. It was not, however, without its advantages. None but men of education concerned themselves with controversy, and hence the boldest, most showy, or most dashing writer, was less likely to teach others, a little younger than himself, to undervalue the long labours and hard-earned discoveries of his predecessors.

"Let us now see the arguments by which Mr. Hunter was convinced, that properties exist in the blood, corresponding to those by which we admit life in the solids.

"His section *on the living principle of the blood* begins with remarking, that the difficulties of admitting life in a fluid, must, he conceives, arise from our always connecting certain ideas of form and organization with life. Yet, as he justly adds, no one is so ignorant of medicine, as not to recollect the stress which is always laid on the appearance of this fluid in disease, and the prognosis we are ready at deriving from such appearances. Must we impute such to the action of vessels on dead matter? That life may exist, where we can neither discover organization nor voluntary motion, he very fairly infers, from the property which the yolk of an egg has to preserve itself from putrefaction, though exposed to a heat of 103° for three or four weeks. That this power depends on life, he inferred, from finding that it exists only in such eggs as are hatched. Others, which never hatched, became putrid about the same time as any dead animal matter, exposed to a similar heat.

"He found also, that a living egg was subject to the same laws, in preserving its temperature, as the less complicated animals. When eels, frogs, or lizards, were exposed to cold, greatly below the freezing point, they could be reduced about two degrees below that point: in this state, they remained for some time, when they suddenly rose to the freezing point, being unable to resist the freezing process, and for the most part died; after which, they were as readily frozen, as any other dead matter. A similar difference he found in eggs. These experiments prove, that a living egg has a power of resisting cold similar to some animals, and that this power is destroyed by a similar effect, from the same cause in each. Dr. Currie also found that blood frozen, under certain circumstances, lost its power of coagulation.

"It has been objected, with truth, that blood has been frozen without the destruction of its coagulating power. This is admitted, and the same has been experimentally proved in other parts of animals. The frost bitten, we know, is not always attended with death; and Mr. Hunter froze the ears of rabbits, after which the parts recovered. He also froze detached pieces of muscle, which afterwards shewed a power of contraction. The circumstances under which a whole animal, or a part, may be frozen, without the destruction of life, are detailed by Mr. Hunter, and appear similar in the blood.

Other experiments proved, that heat would produce the same effect on the blood, when recently taken from the body, as on a portion of muscle cut from an animal recently killed. In both, action was more quickly induced; blood heated to 120, coagulated much earlier than portions of the same blood exposed to the common atmosphere. Portions of recent muscle likewise stiffened earlier, in proportion to the degree of heat to which they were exposed; from which, Mr. Hunter fairly draws the following inference: That the coagulation of the blood, and that contraction of muscles which produces stiffening of the body after respiration has ceased, may be hastened by the same exciting cause; and, consequently, depend on the same principle, namely, on life. It will hereafter be shown, that the loss of this principle prevents coagulation of the blood and also stiffening of the body; but let us first see how far this doctrine of the life of blood is confirmed by its actions in the living body.

As the separation of its part, and coagulation of one part, are the only actions of the blood obvious to our senses, let us trace the few varieties of this action, and the circumstances under which it does not spontaneously take place at all.

The blood is for the most part only fluid when circulating in the vessels. This circulation is absolutely necessary for a substance from which the whole body is formed, and by which it is preserved: and no sooner are its services required for the growth, repair, or preservation of the solids, than the blood separates its solid parts. This is best traced in disease or accident. Under hæmorrhage the blood coagulates with greater rapidity, in proportion to the necessity, if possible, of preserving the animal, by plugging the vessels by means of such coagulum *. Where mortification is approaching, the blood prepares itself to prevent the destruction of the animal, which would follow the loss of all its blood: for this purpose, it coagulates in the vessels themselves; and, where an artery is so far injured that its reparation is not within the power of the economy, a firm coagulum is formed, so as to obliterate the cavity of the vessel †. Under common injuries, hæmorrhage would continue till life is extinguished, if no immediate provision was made. This provision is coagulated blood; and in these cases the blood coagulates with a firmness in proportion to the powers of the animal and the high action excited. For this reason the coagulation is sometimes slower, though the separation of the parts is more rapid; hence the red particles, being the heaviest, subside before the consolidation of the lymph; and the latter being unincumbered by such particles, is much firmer in its texture, exhibiting on its surface almost a membranous appearance. These changes we can trace in the blood taken from the veins; and, if we have an opportunity of examining the injured or inflamed parts, we perceive the advantages derived from such changes in the blood. Large portions of lymph, in a coagulated state, consolidate, by

* See Dr. Jones's most interesting experiments. † Ibid.

its its adhesive property, all the divided parts; inflamed membranes are united to each other in parts before loosely in contact: when this union does not take place, we find the process of inflammation spread along a whole membrane, and its life, and in consequence the life of the animal, destroyed, as happens when the constitution, from any cause, is incapable of exciting this necessary action.

"It may be asked, if all these provisions are made, why does an animal ever bleed to death? or why is an animal destroyed by inflammation? The only answer to this is, that whatever we see in this world is imperfect; and of this we are hourly reminded in the human frame. If the common processes of life are not always unattended with disease, should we wonder if the means of restoration after injury should not be always apportioned to the end, or if death should actually follow the high action excited by the stimulus for preserving a part? To express our surprise at such events is little less absurd, than to inquire what is life. Our only business, and all that we can ever accomplish, is to trace the phenomena of life, under health and under disease, and as far as possible avail ourselves of such knowledge in our attempts to alter or suppress irregular or too high action in the system.

"Let us now consider the circumstances under which the blood never coagulates. These are twofold; the first is, when its coagulation would be injurious to life; the second, when it is incapable of coagulating, from having lost its life.

"The menstrual blood never coagulates; coagulation would be injurious to that necessary process: but in the same parts, under hæmorrhage, coagulation takes place, as a means of lessening the flow of blood. In priapism coagulation never takes place: if it did, it would probably be destructive of the organization of the parts. In torpid animals, when the blood moves so slowly as would be insufficient to prevent coagulation out of the body, the blood never coagulates, or coagulates so slightly as readily to recover its fluidity. In a fainting fit the blood never coagulates. Under all these circumstances, the blood is so far connected with some solid part of the body, or with the whole, as to render its fluidity necessary for the existence of such part, or for the preservation of its functions, or for its future free circulation. Out of the body no such cause can exist, and in consequence we find the first action we discover is, a separation of its parts; the next, coagulation.

"But it has been remarked, that there are circumstances under which no coagulation takes place, even out of the body. The most common cause of this is the death of the blood; and, as Mr. Hunter remarks, it is not easy at all times to ascertain whether the blood or the solid parts die first. In the common modes of killing animals, the muscles still retain the capacity to be stimulated into contraction after respiration has ceased, and the blood also retains its power of coagulation. The last act of the muscles is that by which the body is universally stiffened, and coagulation in in the blood takes place about the same time. In death, after more lingering diseases, the body will scarcely stiffen, and the blood very slightly coagulates. In the worst form of malignant fevers the vessels seem incapable of transmitting the blood in the customary way, and the blood of preserving its life. Hence we discover it in weals or patches, immediately under the skin, of a purple colour; and, if taken from the arm, it shows no power of coagulation, or of acquiring its crimson colour.

"In the most common mode of dying, I have observed that, for some time after respiration has ceased, the various parts retain life, as is proved by the irritability of the muscles, the contraction of the blood-vessels, and the coagulation of the blood. But there are modes of death by which the life of every part seems instantly destroyed. In animals, killed by lightning or electricity, it is found that the body never stiffens, neither the muscles nor blood-vessels contract; and in these cases the blood never coagulates. The same occurs under other modes of death, but not with the same certainty. In death, from violent passions of the mind, from a blow on the stomach, or by a wound in the spine, it will sometimes happen that the body never stiffens; and as often as this is the case the blood never coagulates.

"I have thus gone through the more obvious proofs that the life of the blood depends on a continuance of the same causes as the life of the solid parts. They are, I trust, sufficient to show, that every action we can trace in the blood depends on its life, and that they all cease with its life. But the reader is not to suppose that the proof of this proposition is a matter of mere curiosity. Like the problems of geometry, in the various branches of philosophy, it is to be kept in view in every part of Mr. Hunter's doctrine of inflammation; a doctrine, without the knowledge of which all those judicious observations, which the Reviewer compliments so highly, must be insufficient to direct us in that variety of forms in which inflammation occurs, and often proves fatal before it is suspected. I take no notice of the number of local actions which attend every abscess and every ulcer, which till Mr. Hunter's time were unnoticed, or explained on principles inconsistent with the laws of animal economy, and the practice which was more commonly empirical than scientific."

---

*Biographical Account of Joseph Black, M.D. F.R.S.E. &c. Professor of Chemistry in the University of Edinburgh.* By Thomas Thomson, M.D. F.R.S.

The materials from which the following account was drawn, were first given to the public in the preface to Dr. Black's Lectures, edited by Professor Robison. Mr. Robison informs us that he was indebted for most of his facts "to a paper read to the Royal Society of Edinburgh by the near relation of Dr. Black, Dr. Adam Ferguson, Professor of Mathematics in the University, and well known in the republic of letters by works of the very first rank."

Dr.

Dr. Joseph Black was born in France on the banks of the Garonne in the year 1728. His father, Mr. John Black, was a native of Belfast, in Ireland, but of a Scotch family, which had been some time settled there. Mr. Black resided for the most part at Bourdeaux, where he carried on the wine trade. He married a daughter of Mr. Robert Gordon, of Hilhead, in Aberdeenshire, who was also engaged in the same trade at Bourdeaux.

The mother of Dr. Black, and the mother of Mr. James Russel, Professor of Natural Philosophy in the University of Edinburgh, were sisters; and the mother of Dr. Adam Ferguson was their aunt, a circumstance which was the origin, though not the cement, of a friendship subsisting between them through life.

In the year 1740, young Black, then in the 12th year of his age, was sent to Belfast, that he might have the education of a British subject. After finishing his grammar-school education, he went, in 1746, to the University of Glasgow. Dr. Cullen had commenced his great literary career, and, having made choice of philosophical chemistry as a field still untrodden, was delivering lectures upon that science in the University of Glasgow. These lectures caught the congenial fancy of young Black, who speedily became a zealous chemist, and the favourite pupil and friend of his master. Mr. Black had made choice of medicine as the profession to which he proposed to attach himself; and, in 1750 or 1751, he went to the University of Edinburgh to finish his medical studies. Here he lived in the house of his cousin-german, Mr. James Russel, Professor of Natural Philosophy, in whose society he must have passed his time both agreeably and profitably.

At this period the opinions of the medical professors were divided about the manner in which certain lithontriptic medicines acted in alleviating the excruciating pains of the stone. One of these medicines was lime-water. They all belonged to the class of bodies called caustic, and their efficacy was ascribed to this causticity. Now this causticity was always induced, either directly or indirectly, by the fire. Thus lime-stone, in its natural state, possesses no caustic properties; but, by exposure to a strong heat, it is converted into the caustic substance called quick-lime. The investigation of the nature and cause of this causticity was considered as very important. It drew the particular attention of Mr. Black. He investigated the subject with his accustomed precision and coolness; and, having ascertained it in a satisfactory manner, made it the subject of his inaugural dissertation in 1754, when the degree of Doctor of Medicine was conferred upon him by the University of Edinburgh. Next year he published his experiments upon magnesia alba, quick-lime, and other alkaline substances, in which the whole subject was developed at length.

Just at this time, Dr. Cullen was removed to Edinburgh, and the chemical chair in Glasgow became vacant. Dr. Black's experiments on magnesia and quick-lime, which afforded by far the finest specimen of chemical investigation hitherto offered to the public, secured him that chair. Accordingly he was appointed

Professor of Anatomy, and Lecturer on Chemistry, in the University of Glasgow. Not considering himself as sufficiently qualified for the anatomical class, he exchanged tasks with the Professor of Medicine. While in Glasgow, therefore, his lectures on the institutes of medicine constituted his chief task. He engaged, likewise, in the practice of medicine; and, from the sweetness of his manners, and the goodness of his heart, soon became a favourite practitioner.

While in Glasgow, he brought to maturity his speculations concerning heat, which had occupied his attention from the very commencement of his medical studies.

Dr. Black continued in the University of Glasgow from 1756 to 1766. In that year, Dr Cullen, Chemical Professor in Edinburgh, was appointed Professor of Medicine, and thus a vacancy was made in the chemical chair of that University. There was but one wish with respect to a successor. Indeed, when the vacancy happened in 1756, by the death of Dr. Plummer, Dr. Black's reputation stood so high, that, had it depended on the University, he would have been appointed to the chair. He had now greatly added to his claim of merit by his important discovery of the procedure of Nature in producing fluidity and vapour; and he had acquired the high esteem of every one by the singular moderation and scrupulous caution which marked all his researches. Dr. Black was appointed to the chemical chair of Edinburgh, to the general satisfaction of the public; but the University of Glasgow sustained an irreparable loss. In this new scene his talents were more conspicuous, and more extensively useful. The number of his pupils underwent a progressive and annual increase during the whole time that he was Professor. Many of these pupils were from the workshop of the artist or manufacturer, and had not enjoyed the advantage of a liberal education. Yet such persons, in the opinion of Dr. Black, constituted by no means the least important part of his class. He laboured, therefore, with the greatest assiduity, to bring his lectures to a level with this least informed part of his audience; and thus every year he rendered them more and more elementary. His lectures were always listened to by his audience with inexpressible delight. His voice was low; but sweet and distinct. His language was simplicity itself; but always apposite, and never vulgar. His experimental illustrations were exactly suited to the object in view, and carried full conviction to the mind of the spectator: there was no glare, no parade, no showman exhibition; but an attic elegance and simplicity highly delightful to a refined and cultivated mind. I describe, (says Dr. Thomson,) the lectures such as I listened to them myself, about the year 1796. At that period, Dr. Black's vigour was nearly gone. Indeed, his state of health was such, that he was obliged to employ an assistant to help him in his experiments, and to lecture for him occasionally. But even with all these disadvantages, his lectures made an impression on my mind, which no time can efface.

Dr.

Dr. Black's health had been always delicate. The least exertion brought on a cough, with a spitting of blood. This obliged him to remain a tranquil spectator of the chemical discoveries which were constantly pouring in from all quarters, and to leave it to others to explore the tempting fields which he had first discovered. Towards the latter period of his life, when undue advantages were taken by certain foreigners of the discoveries which he had made, without any acknowledgment of obligations to the original discoverer, he was urged by his friends to lay an historical detail of the whole of his labours before the public. He began this task more than once; but was always obliged to desist almost immediately, in consequence of the illness brought on by this unusual exertion of thought.

By abstaining from all exertion, by living in the most abstemious manner, and by constant, though moderate exercise, he contrived to enjoy an almost uninterrupted, though feeble state of health, and to prolong his life to a considerable old age, happy to the last day, and capable of enjoying the conversation of a few select friends. His only apprehension was that of a long continued sick bed; and this, perhaps, less from any selfish feeling, than from the humane consideration of the trouble and distress to friends; and never was this modest and generous wish more completely gratified. "On the 26th day of November, 1799, and in the 71st year of his age, he expired without any convulsion, shock, or stupor, to announce or retard the approach of death. Being at table with his usual fare—some bread, a few prunes, and a measured quantity of milk diluted with water; and having the cup in his hand, when the last stroke of his pulse was to be given, he set it down on his knees, which were joined together, and kept it steady with his hand in the manner of a person perfectly at ease. In this attitude he expired, without spilling a drop, and without a writhe on his countenance; as if an experiment had been required to shew to his friends the facility with which he departed. His servant opened the door to tell him that some one had left his name, but, getting no answer, stepped about half way towards him, and seeing him sitting in that easy posture, supporting his bason of milk with one hand, he thought that he had dropped asleep, which he had sometimes seen happen after his meals. He went back and shut the door; but before he got down stairs some anxiety, which he could not account for, made him return and look again at his master. Even then he was satisfied, after coming pretty near him, and turned to go away; but, again returning, and coming quite close to him, he found him without life.

"So ended a life which had passed in the most correct application of reason and good sense to all the objects of pursuit which Providence had prescribed to his lot, with many topics of agreeable recollection, and few things to ruffle his thoughts. He had long enjoyed the tender and affectionate regard of parents whom he loved, honoured, and revered; with the delightful consciousness of being a dutiful son, and being cherished as such—of a family remarkable markable for sweetness of disposition and manners, he had lived with his brothers and sisters in terms of mutual love and attachment. He had never lost a friend, but by the stroke of mortality, and he felt himself worthy of that constancy of regard. He had followed a profession altogether to his taste in a manner which procured him the esteem and respect of all competent judges, and set his name among the most eminent. He was conscious that his reputation was not unmerited; his success and emolument secured the respect even of the ignorant; gave him the command of every rational gratification, and enabled him to add greatly to the comforts of the numerous descendants of his worthy parents—heirs, not only of their name, but likewise of their unambitious moderation and amiable simplicity of character."

The publications of Dr. Black (omitting his inaugural dissertation) amount only to three short papers; for, respecting his discoveries and speculations concerning heat, he published no account whatever. In the year 1803, or three years after his death, his lectures were published by Dr. John Robison, Professor of Natural Philosophy in the University of Edinburgh, who undertook the task of editor at the request of Dr. Black's friends. They appear to me to be a pretty fair representation of his lectures such as they were about the year 1796; and, though it was by no means doing justice to Dr. Black to give to the world lectures never intended for publication, and just at a period too when the science had assumed a new form, quite different from that under which he had been accustomed to view it; yet they convey a very happy view of the elegant simplicity of manner by which he was distinguished, of the very apt illustrations by which the different subjects that he treated were elucidated, and of the true philosophical caution with which all his conclusions were drawn.

His Experiments upon Magnesia Alba, Quick-lime, and other Alkaline Substances, were published in 1755, and constitute, in my opinion, one of the very best specimens of analytical investigation ever offered to the public. It was known, that when limestone is exposed to a violent heat for a sufficient time, it is converted into quick-lime, a substance which has a strong caustic taste, gives a green colour to vegetable blues, corrodes animal and vegetable substances, &c. If pearl-ash be mixed with three or four times its weight of quick-lime, and agitated for some hours in a quantity of water, the quick-lime is converted into chalk, and the pearl-ash becomes exceedingly caustic and corrosive. These facts were sufficiently known; but no satisfactory explanation of them had been offered, though it was generally believed, that the fire had imparted something to the lime to which it was indebted for its causticity. Dr. Black demonstrated, by the most decisive experiments, that lime-stone is a compound of two substances; namely, quick-lime and a peculiar species of air which acts the part of an acid, and to which he gave the name of *fixed air*. The fire disengages this air, and drives it off. The quick-lime remains in a state of purity, and exhibits its natural caustic proper-

 ties.

ties. Pearl-ash is in like manner a compound of pure potash and fixed air. Quick-lime has a stronger affinity for fixed air than potash has.

Such is the outline of the theory which Dr. Black establishes in his dissertation. He establishes likewise the peculiar properties of magnesia, and shews that it differs from every other earthy body.

Dr. Black's speculations respecting heat have had such an effect upon the progress of the science, that it would be unpardonable not to notice them here. A very good account of them will be found in the first volume of his lectures. Indeed, his lectures on heat constituted the most finished, and by far the most valuable, part of his course. It is well known, that the freezing point of water is 32°, that whenever the thermometer sinks below 32° water begins to freeze, and whenever it rises above 32° ice and snow begin to melt. At the first view of the subject, one would be disposed to expect that, as soon as the thermometer sinks below 32°, the whole water would immediately become ice, and that when it rises above 32°, the ice would be as speedily converted into water; but every body knows that these speedy changes never take place. In cold weather a crust of ice is formed upon the surface of rivers and lakes; and, if the cold continue, this crust becomes gradually thicker. But, unless the water be very shallow, it is very seldom or never totally converted into ice. The warm weather returns while a considerable portion of the water of the lake is still unfrozen. We remark the same slowness in the conversion of ice into water. When snow is accumulated in great quantities in mountainous countries, it resists the united action of the sun and the wind for weeks, or even months. It is always melting, indeed, but it melts very slowly; and in some cases the cold weather returns again before the liquefaction is completed. Such were the facts which had been obvious to all the world from the beginning. Dr. Black was the first person who examined them closely and endeavoured to explain them. According to him, water is a compound of two substances—ice and heat. It cannot freeze or be converted into ice till it has parted with its heat; and, as the heat makes its escape but slowly, the water freezes but slowly. Ice, on the other hand, can only be converted into water by combining with a certain quantity of heat; and, as this combination takes place but slowly, the ice melts but slowly. This view of the subject, Dr. Black confirmed by simple and satisfactory experiments. The heat which thus renders water fluid, he called latent heat, because its presence is not indicated by the thermometer. He shewed that the latent heat of water is 140°. He ascertained, likewise, that fluidity in all cases is owing to the combination of latent heat with the body becoming fluid.

It is well known that water and other liquids, when exposed to heat, increase in temperature till they become boiling hot, but after that their temperature remains stationary. They gradually, indeed, boil away, and are converted into steam or vapour, an elastic fluid possessing many of the properties of air; with this difference, difference, that when exposed to the action of cold it is again converted into the very liquid from which it was originally produced. Dr. Black shewed that vapour or steam is a combination of the liquid from which it was produced and latent heat. The latent heat of the vapour of water or steam he found about 950°. It was this doctrine respecting the nature of steam that led Mr. Watt to his great improvements on the steam-engine—improvements which have been of incalculable benefit to the manufactures of Great Britain.

Dr. Black was the first person who pointed out that every substance is possessed of a peculiar specific heat, or that different bodies have different capacities for heat. This subject was afterwards further investigated by Dr. Irvine, of Glasgow, and by Mr. Wilcke, of Stockholm.

A very short paper by Dr. Black was published in the 65th volume of the Philosophical Transactions, for the year 1775, giving an account of some experiments, shewing that recently boiled water begins to freeze more speedily than water that has not been boiled. He found, that if the unboiled water be continually stirred, it begins to freeze as soon as the boiled water. He gives the following explanation of the phenomenon. Water, by boiling, is deprived of a portion of air. When exposed to the atmosphere, it begins to absorb this air, and continues to do so till it has recovered its original quantity. This absorption produces a disturbance in the water, not indeed sufficient to be perceived by the eye, but sufficient to prevent it from becoming colder than 32°, without beginning to freeze.

The only other paper, written by Dr. Black, was published in the second volume of the Transactions of the Royal Society of Edinburgh. It is an analysis of the Geyzer and Rikum springs in Iceland. A quantity of the water of these springs was brought from Iceland by Sir John Thomas Stanley, and sent to Dr. Black. This paper may be taken as a model of the proper manner of examining mineral waters. The following were the constituents found in 10,000 grains of each of these waters:—

| | Rikum. | Geyzer. |
|---|---|---|
| Soda | 0·51 gr. | 0·95 gr. |
| Alumina | 0·05 | 0·48 |
| Silica | 3·73 | 5·40 |
| Common Salt | 2·90 | 2·46 |
| Sulphate of Soda | 1·28 | 1·46 |
| | 8·47 | 10·75 |

*Annals of Philosophy.*

CRITICAL

## Figures and Tables

**Figure f1:**